# Synthesis of Li_6.4_La_3_Zr_1.4_Ta_0.6_O_12_-Incorporated Composite Gel Electrolytes via Competitive Anion Anchoring for Dual-Interface Stabilization in Lithium Metal Batteries

**DOI:** 10.3390/gels12040283

**Published:** 2026-03-28

**Authors:** Jie Zhao, Maoyi Yi, Chunman Zheng, Qingpeng Guo

**Affiliations:** College of Aerospace Science and Engineering, National University of Defense Technology, Changsha 410073, China; zhaojie15@nudt.edu.cn (J.Z.); yimaoyinudt@nudt.edu.cn (M.Y.)

**Keywords:** solid-state battery, composite gel electrolyte, competitive anion anchoring, extreme-condition operation

## Abstract

The demand for high-energy-density and fast-charging solid-state lithium metal batteries (SSLMBs) often subjects practical devices to internal thermal loads, making high-temperature operation a common operational condition rather than an isolated scenario. To address the interfacial degradation and dendrite growth accelerated by such thermomechanical stresses, we developed a composite gel electrolyte (CGE) by incorporating an optimal concentration of active Li_6.4_La_3_Zr_1.4_Ta_0.6_O_12_ (LLZTO) into a fluoropolymer network. The abundant Lewis acidic sites on the LLZTO surfaces promote competitive solvation decoupling by interacting with anions, thereby modulating the primary solvation sheath of Li^+^. This localized modulation lowers the lithium-ion migration activation energy to 0.248 eV and facilitates a dual-interfacial passivation mechanism. Specifically, a rigid, inorganic-rich solid electrolyte interphase (SEI) forms to suppress morphological instability at the lithium anode, while an organic-dominated cathode electrolyte interphase (CEI) enhances the oxidative stability up to 4.3 V. As a result, symmetric cells demonstrate stable electrodeposition for over 450 h at 80 °C and 0.5 mA cm^−2^. Furthermore, NCM811/Li full cells utilizing this CGEs exhibit significantly improved thermal resilience and cycling stability.

## 1. Introduction

Solid-state lithium metal batteries offer high energy density, but their practical operation is often constrained by thermal–electrochemical stresses and the challenge of low ion-transport efficiency at room temperature [1,2,3,4]. Fast-charging protocols generate substantial Joule heating, elevating local temperatures to 60–80 °C [5,6,7]. Although this thermal environment is required to enhance the sluggish chain dynamics of polymer-based electrolytes, it simultaneously accelerates battery degradation [8,9,10]. Thermally softened polymer matrices are susceptible to localized mechanical stresses induced by inhomogeneous lithium electrodeposition, leading to the formation of crack networks [11,12,13]. These cracks allow free anions to continuously react with the lithium anode, forming a resistive, organic-rich interphase that accelerates dendrite growth and capacity fade [14,15,16,17]. Therefore, for solid-state lithium metal batteries operating under practical thermal conditions, improving ionic conductivity alone is not sufficient; the electrolyte must also simultaneously maintain thermomechanical integrity and interfacial stability.

To mitigate these heat-induced failures, traditional strategies rely on adding inorganic ceramic fillers for passive mechanical reinforcement [18,19,20,21,22]. Early studies have shown that inorganic silicate/clay fillers can do more than mechanically reinforce polymer electrolytes. Kurian et al. demonstrated polymer–silicate nanocomposite electrolytes with composition-dependent mechanical and ionic-transport properties, while Lutkenhaus et al. revealed that structurally ordered polymer-clay assemblies could induce anisotropic ion transport, highlighting the important role of polymer/filler interactions in regulating electrolyte functionality [23,24]. These studies suggest that inorganic fillers can actively influence electrolyte microstructure and ion-transport pathways, rather than serving only as inert reinforcing agents. However, excessive filler loading frequently leads to severe agglomeration and high solid–solid interfacial impedance, failing to stabilize electrode interfaces under sustained thermal loads [8,11,12,25,26]. As a result, conventional filler-reinforcement strategies often improve mechanical strength at the expense of transport uniformity and interfacial compatibility. Resolving this limitation requires shifting from simple physical blending to a tailored structural design that modulates local ion solvation dynamics, thereby customizing the passivation chemistry at both electrodes [27,28,29,30]. Such a design is particularly necessary for wide-temperature lithium metal batteries, where fast ion transport, thermal tolerance, and durable electrode/electrolyte interfaces must be achieved simultaneously.

Herein, we report CGEs prepared by incorporating an optimal concentration (0.1 g) of LLZTO into baseline gel electrolytes (GEs) comprising a Poly(vinylidene fluoride-co-hexafluoropropylene) (PVDF-HFP) framework and a 1-Ethyl-3-methylimidazolium bis (trifluoromethylsulfonyl) imide (EMITFSI) ionic liquid. This design utilizes the Lewis acid sites on the LLZTO surface to induce competitive anion anchoring within the polymer–ionic liquid matrix [29,31]. This interaction effectively dissociates bulky TFSI^−^ anions from the primary solvation sheath of Li^+^, lowering the lithium-ion migration activation energy to 0.248 eV while ensuring robust mechanical stability (a tensile strength of 1.78 MPa and an elongation at break of >200%). Furthermore, this modulated ion flux facilitates a targeted dual-interfacial chemistry: a dense, inorganic-rich SEI that suppresses anodic dendrites, and a conformal, organic-dominated CEI. By coupling microscale solvation regulation with macroscopic thermomechanical stability, this CGE enables stable, polarization-free cycling for over 450 h at 80 °C, providing a viable strategy for high-temperature solid-state energy storage. More importantly, the significance of this work lies in demonstrating that LLZTO can function not merely as a passive filler, but as an active inorganic component that regulates ion solvation, interfacial chemistry, and mechanical stability in a coordinated manner. This makes the present design both scientifically meaningful for understanding polymer/inorganic hybrid electrolytes and practically important for developing reliable wide-temperature solid-state lithium metal batteries.

## 2. Results and Discussion

### 2.1. Characterization of Electrolytes

**Figure 1:**
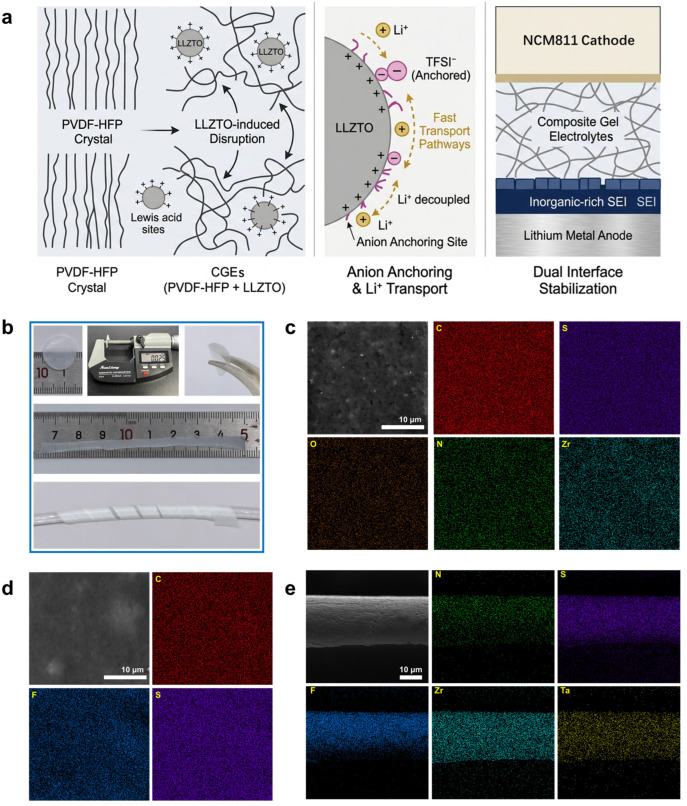
(**a**) LLZTO-induced crystallinity disruption, competitive anion anchoring, and dual-interface stabilization in CGEs. (**b**) Optical photograph of the CGE film. (**c**) SEM image and corresponding elemental mapping images of CGEs with 0.1 g LLZTO loading. (**d**) SEM image and corresponding elemental mapping images of GEs. (**e**) Cross-sectional SEM images and corresponding elemental mapping images of CGEs with 0.1 g LLZTO loading.

The GEs and a series of CGEs with varying LLZTO contents were prepared via a solution-casting method (Figure 1b and Figure S1). The pristine GEs exhibit a transparent and flat appearance, whereas the LLZTO-incorporated CGEs are translucent and white. Both electrolytes form uniform films with a thickness of approximately 25 μm. At the cell level, this reduced thickness decreases the mass and volume fractions of inactive components, which is beneficial for improving energy density. Furthermore, it shortens the Li^+^ transport distance, helping to mitigate polarization. As shown in Figure 1b, the CGE membrane exhibits good mechanical flexibility and self-supporting capability. It can withstand repeated folding and be wrapped tightly around a thin glass rod without observable macroscopic fracture. This structural compliance suggests adaptability to the winding processes required for pouch cells and the ability to buffer electrode volume fluctuations during cycling, thereby maintaining continuous solid–solid contact at the interfaces.

The microstructural and compositional distributions were examined using scanning electron microscopy (SEM) and energy-dispersive X-ray spectroscopy (EDS). The unmodified GEs (Figure 1d and Figure S2) present a dense, continuous polymer matrix with a homogeneous distribution of C, F, and S, indicating good compatibility among the PVDF-HFP, ionic liquid (IL), and lithium salt. With the introduction of LLZTO (Figure 1c,e), the CGEs maintain a dense morphology while integrating the inorganic and organic phases uniformly. EDS mapping (Figure 1c and Figure S3) confirms that the flexible constituent elements (C, N, S, O) spatially overlap with the Zr signal from the LLZTO particles. This demonstrates that the nanoscale LLZTO is homogeneously dispersed within the polymer phase, avoiding stress concentration defects typically caused by particle agglomeration. Moreover, cross-sectional SEM and EDS analyses (Figure 1e and Figure S4) reveal an even distribution of Zr and Ta along the thickness direction, without visible micro-voids or particle sedimentation. This bulk homogeneity provides the structural basis for a continuous Li^+^ conductive network.

The impact of active filler concentration on the microstructural integrity of the CGEs was further investigated (Figures S5–S10). At low concentrations (0.05–0.1 g), LLZTO particles are homogeneously dispersed. However, exceeding an optimal loading threshold leads to severe agglomeration, where nanoparticles form micrometer-scale clusters. This structural distortion reduces the effective interfacial area and compromises the continuity of the polymer matrix. Kinetically, these agglomerates can act as barriers that obstruct long-range Li^+^ transport pathways, increasing local mass-transfer resistance. Mechanically, the clustered regions create stress concentration points. Under the mechanical stress of lithium dendrite growth, these weak points can initiate cracking within the polymer matrix, potentially leading to cell short-circuits. Therefore, optimizing the inorganic filler loading is critical to balancing polymer flexibility, inorganic phase dispersion, and overall structural integrity.

### 2.2. Characterization of Properties of Electrolytes

**Figure 2:**
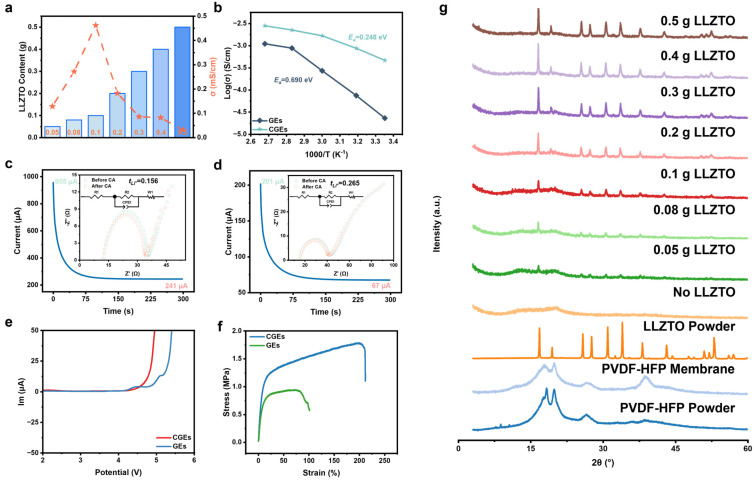
(**a**) The room-temperature ionic conductivities of the CGEs with different LLZTO contents. (**b**) Arrhenius plots of ionic conductivity for GEs and CGEs. (**c**) Chronoamperometry profile of the Li/GE/Li symmetric cell and the applied DC potential of 20 mV (inset: corresponding Nyquist plots before and after polarization). (**d**) Chronoamperometry profile of the Li/CGE/Li symmetric cell and the applied DC potential of 20 mV (inset: corresponding Nyquist plots before and after polarization). (**e**) Linear sweep voltammetry curves of GEs and CGEs. (**f**) Stress–Strain Curve of GEs and CGEs. (**g**) XRD patterns of pristine PVDF-HFP, LLZTO powder, and CGEs with varying LLZTO loading.

To determine the optimal loading of the inorganic filler and its effect on ion transport kinetics, electrochemical impedance spectroscopy (EIS) was performed on CGEs with LLZTO contents ranging from 0.05 to 0.5 g. As shown in Figure 2a and Figure S11, the room-temperature ionic conductivity initially increases with ceramic loading, reaching a maximum of 0.46 mS cm^−1^ at an optimal LLZTO content of 0.1 g. This enhancement is attributed to the interfacial interactions between the Lewis acidic sites on the LLZTO surfaces and the polymer segments, which diminish the overall mass-transfer resistance. However, at higher concentrations (0.2 to 0.5 g), the ionic conductivity significantly decreases. Excessive inorganic loading leads to particle agglomeration, which disrupts the continuous Li^+^ conducting polymer network. The resulting steric hindrance at the solid–solid interfaces restricts the segmental motion of the flexible polymer chains and obstructs long-range ion transport pathways. Consequently, all subsequent electrochemical evaluations were conducted using the CGEs with 0.1 g LLZTO.

This macroscopic transport behavior is further supported by microstructural analysis using X-ray diffraction (XRD, Figure 2g). The pristine PVDF-HFP matrix exhibits typical crystalline diffraction peaks at 2θ = 18°, 20°, 26°, and 38°. With the addition of the lithium salt and coordinating solvent, these crystalline features are significantly attenuated, leaving a broad peak around 20° that indicates the presence of residual crystalline domains. At the optimal loading of 0.1 g, the system largely maintains an amorphous structure, which suggests an increased free volume fraction, while displaying distinct diffraction peaks corresponding to the cubic garnet phase. This structural state indicates that the homogeneously dispersed LLZTO nanoparticles suppress the recrystallization of the fluorinated polymer chains through interactions between their surface Lewis acidic sites and the polymer matrix. In contrast, at higher concentrations (≥0.2 g), the intensity of the LLZTO crystalline peaks increases prominently.

The optimized structural integration fundamentally improves the ion-transport thermodynamics of the system. According to the Arrhenius fitting curves (Figure 2b), the pristine GEs exhibit a high ionic migration activation energy (*Ea*) of 0.690 eV, indicating that Li^+^ transport is constrained by intricate interactions with highly polar polymer segments and un-dissociated anion clusters. In the CGE network, the activation energy is significantly reduced to 0.248 eV. This reduction suggests that the Lewis acid sites at the LLZTO interfaces induce competitive solvation decoupling, effectively lowering the thermodynamic energy barrier associated with the frequent coordination–dissociation cycles of Li^+^. Consequently, low-barrier ion transport is facilitated along the continuous organic–inorganic interfaces.

Furthermore, the solvation decoupling effect promotes the selective transport of Li^+^. Chronoamperometry measurements (Figure 2c,d) show that under an applied polarizing electric field, the current of the pristine GEs decays from 955 μA to 241 μA, corresponding to a low lithium-ion transference number ($t_{Li^{+}}$) of 0.156. This indicates that free anions dominate the ion transport process. In contrast, the CGEs exhibit a more stable polarization response, with the current decreasing from 201 μA to 67 μA, resulting in an enhanced $t_{Li^{+}}$ of 0.265. This improvement is attributed to the anion-trapping effect of the organic–inorganic network, where the LLZTO particles chemically anchor the mobile anions, thereby facilitating the selective transport of Li^+^.

The enhanced oxidative stability of the composite interface is confirmed by the linear sweep voltammetry (LSV) results (Figure 2e). The unmodified baseline polymer network undergoes anodic oxidative decomposition at approximately 4.1 V (vs. Li/Li^+^). Conversely, the electrochemical stability window of the CGE system is broadened to ~4.3 V. This improved high-voltage tolerance is primarily derived from the introduction of wide-bandgap inorganic fillers and the immobilization of anions via strong intermolecular forces. Such interfacial stabilization effectively mitigates parasitic degradation reactions at the cathode side, demonstrating that the optimal CGE composition balances rapid ion conduction with high-voltage compatibility.

Beyond electrochemical performance, the mechanical properties of the electrolytes were evaluated to determine their practical viability. Uniaxial tensile tests reveal that the addition of LLZTO overcomes the conventional trade-off between stiffness and ductility. The pristine GEs show a tensile strength of 0.92 MPa and an elongation at break of 81.44%. In contrast, the CGEs demonstrate a concurrent enhancement, with both mechanical strength and ductility increasing significantly to 1.78 MPa and over 200%, respectively. This toughening mechanism is attributed to the reconstruction of microscale stress-transfer pathways, in which the homogeneously dispersed LLZTO nanoparticles act as rigid nodes that bear mechanical strain and retard microcrack propagation. Ultimately, the high tensile strength builds a robust barrier against lithium dendrite penetration, while the high elongation capacity ensures continuous and conformal interfacial adhesion during repeated Li plating/stripping cycles, effectively preventing cell impedance increase and internal short-circuits.

**Figure 3:**
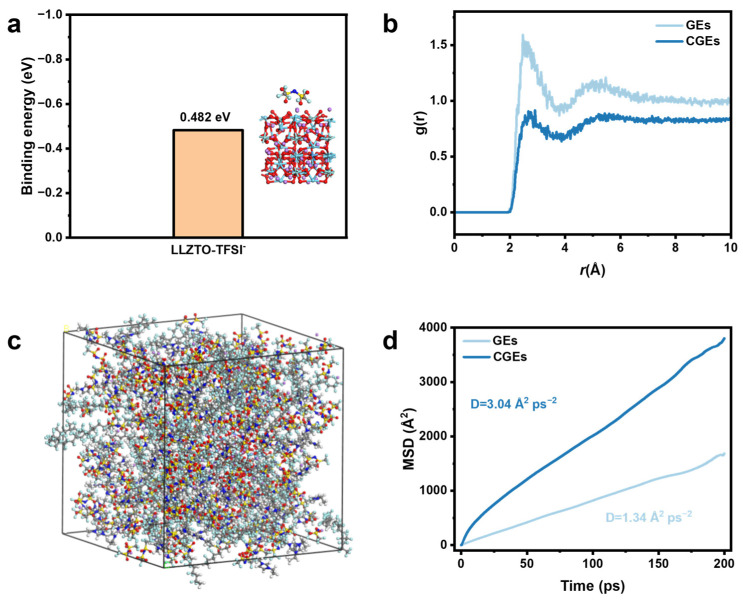
(**a**) Binding energy of LLZTO-TFSI^−^. (**b**) RDF profiles of Li^+^ and O interaction. (**c**) Kinetic model of CGEs. (**d**) MSD trajectories of Li^+^ as a function of simulation time over a 200 ps relaxation window.

Density functional theory (DFT) calculations and molecular dynamics (MD) simulations were executed to elucidate the regulatory mechanisms of the LLZTO inorganic fillers on the internal ionic coordination environment and transport kinetics. Figure 3a illustrates the calculated binding energy between the LLZTO surface and TFSI^−^, where the adsorption energy of 0.482 eV signifies a strong affinity primarily driven by the interaction between the Lewis acidic sites of the ceramic and the electronegative atoms of the anions. This localized surface adsorption provides a fundamental thermodynamic basis for the competitive anchoring of TFSI^−^ at the organic–-inorganic interface.

The influence of this interfacial anchoring on the local solvation configurations was further characterized by the radial distribution function (RDF) segments derived from the MD structural models in Figure 3c and Figure S12. Figure 3b displays the RDF curves for the interactions between Li^+^ and the O atoms of the TFSI^−^ anions. Within the first coordination shell between 2.0 and 3.0 Å, the pristine GEs exhibit a sharp and high-intensity peak, which indicates that a significant fraction of Li^+^ remains coupled within contact ion pairs or larger clusters due to insufficient salt dissociation in the pure polymer network. Upon the introduction of LLZTO, the intensity of this primary coordination peak undergoes a substantial reduction and the subsequent coordination features become increasingly diffuse. This structural evolution suggests that the strong LLZTO-TFSI^−^ binding energy effectively promotes the dissociation of LiTFSI by pulling the anions toward the filler surface while simultaneously liberating Li^+^ from the anion-dominant solvation sheath.

The transition from microscopic solvation decoupling to macroscopic ion transport was further quantified using mean square displacement (MSD) trajectories, as shown in Figure 3d. In the pristine GE network, the Li^+^ MSD increases slowly over the 200 ps simulation and yields a low diffusion coefficient of 1.34 Å^2^ ps^−1^. This restricted mobility is attributed to the strong electrostatic dragging forces and the high activation energy of 0.248 eV required for Li^+^ to hop between coordination sites. Conversely, the CGE system exhibits a rapid linear increase in MSD with a calculated diffusion coefficient of 3.04 Å^2^ ps^−1^. This significant enhancement in transport kinetics is consistent with the experimental improvements in ionic conductivity and lithium transference numbers. The simulation snapshots suggest that the liberated Li^+^ monomers can efficiently percolate through the continuous and high-speed transport pathways established along the inorganic–polymer interphases. These computational findings collectively provide a molecular-level validation of how surface-chemical interactions dictate the bulk electrochemical performance of the composite electrolyte system.

### 2.3. Investigation of the Interfacial Compatibility Between Electrolytes and the Lithium Anode

**Figure 4:**
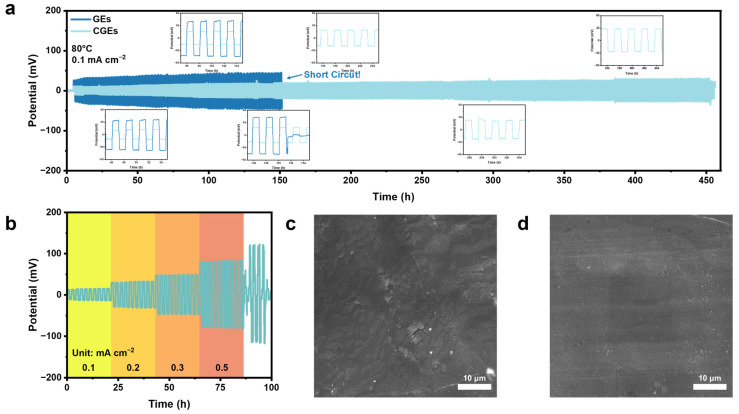
(**a**) Long-term cycling tests of GEs and CGEs at 80 °C and 0.1 mA cm^−2^. (**b**) Galvanostatic lithium plating/stripping tests of GEs and CGEs at different current densities. (**c**) SEM image of the Li metal surface from the cycled symmetric cell using the GEs. (**d**) SEM image of the Li metal surface from the cycled symmetric cell using the CGEs.

As depicted in Figure 4a, under a constant current density of 0.1 mA cm^−2^, the baseline GEs initially exhibit a stable voltage plateau. However, the overpotential subsequently increases, leading to a sudden voltage drop after approximately 150 h. This cell failure is primarily attributed to the irreversible accumulation of inactive dead lithium and the penetration of lithium dendrites across the electrolyte. In contrast, the LLZTO-integrated CGE demonstrates enhanced interfacial stability. Instead of exhibiting progressive polarization, it maintains a low overpotential and displays highly symmetrical voltage profiles over an extended 450 h operational period. A magnified inset further confirms that the plating/stripping voltage profiles retain their rectangular shape throughout the test, with no discernible signs of progressive polarization caused by interfacial side reactions.

To evaluate the current-handling capability of the system, a stepwise critical current density test was performed (Figure 4b). The pristine GEs exhibit kinetic limitations, sustaining stable Li plating/stripping only below 0.2 mA cm^−2^. When the current density is increased to 0.3 mA cm^−2^, noticeable high polarization occurs, indicating that the intrinsic ion transport kinetics are insufficient to accommodate the local charge transfer demands at the electrode/electrolyte interface. Conversely, the CGE demonstrates improved electrochemical stability, delivering a highly reversible plating/stripping response with low polarization even at a higher current density of 0.5 mA cm^−2^. This improvement arises from a synergistic mechanism: the high-modulus LLZTO scaffold forms a rigid network that physically suppresses dendrite penetration, while its surface-exposed Lewis-acidic sites chemically immobilize TFSI^−^ anions. This homogenizes the local Li^+^ flux and reduces localized electric-field concentrations at the interface, thereby inhibiting heterogeneous dendrite nucleation. The mechanical integrity of the ceramic framework prevents deformation-induced cracks, and the anion-anchoring effect ensures uniform current distribution, enabling the system to operate stably under conditions where conventional polymer electrolytes typically fail.

Morphological analysis provides further structural evidence for these electrochemical behaviors. SEM images (Figure 4c) reveal that the lithium electrode paired with the GEs is covered with a porous, moss-like layer of dead lithium. This indicates the continuous consumption of electrolytes at the reactive interface, leading to subsequent morphological degradation. In contrast, the lithium electrode cycled with the CGEs (Figure 4d) remains dense and smooth, without observable microcracks or irregular topographic features. This demonstrates that the composite electrolyte effectively suppresses stress-induced cracking and non-uniform lithium deposition, thereby preserving the structural integrity of the interface over long-term cycling.

To investigate the interfacial stabilization mechanism of the CGEs, depth-resolved X-ray photoelectron spectroscopy (XPS) with sequential Ar^+^ sputtering was performed on the Li metal electrodes after 50 galvanostatic cycles. This enabled the chemical profiling of the SEI.

**Figure 5:**
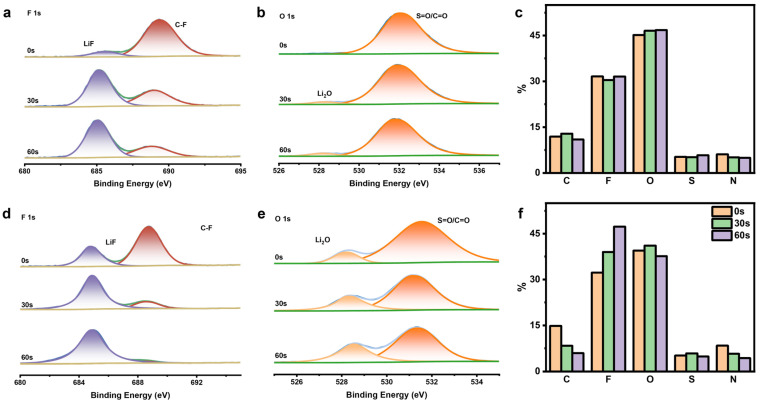
F 1s XPS spectra (**a**) and O 1s XPS spectra (**b**) at various sputtering depths of the Li metal surface from the long-term-cycled Li/GE/ Li symmetric cell. (**c**) Elemental distributions for Li metal by etching times from the cycled symmetric cell using the GEs. F 1s XPS spectra (**d**) and O 1s XPS spectra (**e**) at various sputtering depths of the Li metal surface from the long-term-cycled Li/CGE/ Li symmetric cell. (**f**) Elemental distributions for Li metal by etching times from the cycled symmetric cell using the CGEs.

In the CGE-derived SEI (Figure 5d,e and Figure S13), the outermost layer (0 s sputtering) is predominantly composed of organic species, indicated by C 1s, F 1s, and N 1s peaks, which correspond to the partial decomposition products of PVDF-HFP, EMI^+^ and TFSI^−^. As sputtering progresses to deeper layers (30 s and 60 s) near the Li metal side, the intensity of these organic signatures gradually decreases, while the signals of inorganic constituents steadily increase. Specifically, the electrochemical decomposition of PVDF-HFP, EMI^+^ and TFSI^−^ engenders an abundance of inorganic passivating species, including LiF, Li_2_O, Li_2_S, and Li_3_N. This demonstrates that the CGEs enable the in situ formation of an inorganic-rich SEI during cycling. Corroborated by the aforementioned SEM morphological observations, this well-formed SEI is exceptionally uniform and dense. Furthermore, the quantitative elemental profiling (Figure 5f) corroborates a well-defined organic-rich outer and inorganic-rich inner gradient architecture, unequivocally indicating that this robust SEI is highly effective in mitigating the continuous parasitic decomposition between the electrolyte and lithium metal.

This hierarchical structure is attributed to the presence of the LLZTO, which competitively anchors residual anions at the inorganic–polymer interface, thereby reducing continuous polymer reduction. Near the Li metal surface, the concentrated Li^+^ flux drives the targeted electrochemical reduction in the constrained anions, reinforcing the dense inorganic sublayer rich in LiF, Li_2_O, Li_3_N, and Li_2_S. This inorganic-dominant inner layer provides sufficient mechanical stiffness to suppress dendrite penetration while facilitating high Li^+^ conductivity, serving as a dual-functional barrier that dramatically improves long-term interfacial stability.

By contrast, the SEI formed in the GEs shown in Figure 5a–c and Figure S14 exhibits a markedly different chemical composition and structural evolution. Although the F 1s spectra indicate the presence of LiF, which likely originates from the limited decomposition of TFSI^−^ anions or the PVDF-HFP matrix, the critical passivating constituents, including Li_2_O and Li_3_N, are conspicuously absent throughout the entire depth profile. The C 1s and O 1s signals persistently dominate the spectra because the high carbon and oxygen content exceeding 70% is primarily attributed to the pervasive accumulation of undecomposed or partially decomposed polymer fragments and salt anions.

Unlike the CGEs that develop a multi-component gradient, the SEI formed in the GEs maintains a remarkably uniform but inorganic-deficient composition from the surface to the bulk interphase (Figure 5c). This single-phase-dominant chemistry lacks the structural support of oxide and nitride species and indicates that the baseline electrolyte undergoes continuous and uncontrolled decomposition without achieving effective self-passivation. When coupled with the previous SEM findings, it is evident that such an SEI is morphologically inhomogeneous and porous. Consequently, it fails to provide a sufficiently robust barrier to isolate the reactive lithium metal and leads to persistent electrolyte consumption as well as accelerated interfacial failure during cycling.

### 2.4. Performance Validation and Mechanistic Analysis of Electrolytes in Solid-State Batteries

To assess the practical viability of the CGEs, full cells involving either high-voltage Ni-rich LiNi_0.8_Co_0.1_Mn_0.1_O_2_ (NCM811) or olivine-type LiFePO_4_ (LFP) cathodes were assembled and evaluated at 80 °C.

**Figure 6:**
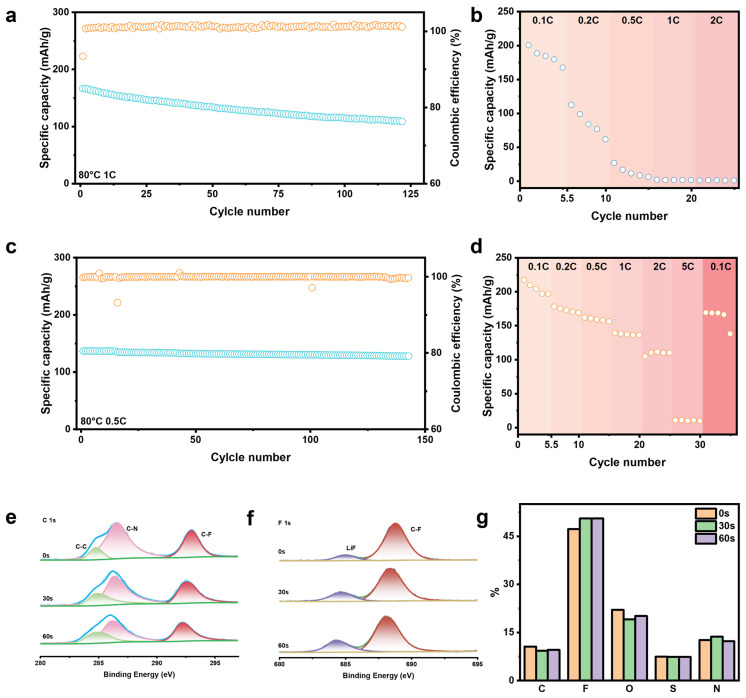
(**a**) Cycling performance of the NCM811/CGE/Li cell. (**b**) Rate capability of the NCM811/GE/Li cell. (**c**) Cycling performance of the LFP/CGE/Li cell. (**d**) Rate capability of the NCM811/CGE/Li cell. C 1s XPS spectra (**e**) and F 1s XPS spectra (**f**) at various sputtering depths of the cycled NCM811 cathode. (**g**) Elemental distributions for the cycled NCM811 cathode.

As illustrated in Figure 6a and Figure S15, the NCM811/CGE/Li cell retains 65.8% of its capacity after 120 cycles at 1 C and 80 °C, with an average Coulombic efficiency of >99.5%. This indicates enhanced thermal and oxidative stability, which suppresses parasitic side reactions at the highly reactive Ni-rich interface. Identical testing with the LFP cathode (Figure 6c and Figure S16) shows that under 0.5 C cycling at 80 °C, the cell retains 93.4% of its initial capacity over 150 cycles, with an average Coulombic efficiency of 99.87%. The consistent performance across different cathode chemistries confirms that the enhancement originates from the intrinsic interfacial regulation of the CGE.

The rate capability tests (Figure 6b,d) further corroborate this kinetic advantage. While baseline GE-based cells undergo rapid capacity decay beyond 0.2 C and fail to function stably at 1 C, the CGE-enabled cell delivers a capacity of 111.1 mAh g^−1^ at a higher rate of 2 C. When the current is reverted to 0.1 C, the capacity recovers to 169mAh g^−1^. This high reversibility and low polarization indicate that the CGE preserves stable electrode kinetics under high-current operation.

To further elucidate the interfacial chemistry, XPS was conducted on the cycled NCM811 cathode (Figure 6e–g and Figure S17). In contrast to the inorganic-rich SEI observed at the anode, the CEI exhibits an organic–inorganic composite structure. The partial oxidative decomposition of the PVDF-HFP chains and TFSI^−^ anions lead to the formation of LiF. Concurrently, the C 1s, N 1s, and S 2p spectra indicate the retention of undecomposed PVDF-HFP, EMI^+^, and TFSI^−^ species. Together, these components form an “organic-dominant with inorganic-assisted” CEI network. Elemental depth profiling (Figure 6g) confirms that the composition remains largely constant throughout the interphase layer. Mechanistically, the flexible polymer matrix accommodates volume-fluctuation-induced strain during (de)lithiation, while the robust LiF component preserves the thermodynamic stability of the high-voltage interface. Ultimately, this flexible CEI pairs with the rigid SEI at the anode to establish a complementary interfacial framework that supports long-term full-cell operation.

Collectively, these findings establish that the incorporation of the LLZTO goes beyond passive mechanical reinforcement. Through multiscale Lewis acid–base interactions, it restructures the dual-interfacial chemistry: inducing a high-modulus, inorganic-rich SEI at the lithium anode, while simultaneously architecting a flexible, organic–inorganic CEI at the cathode. This synergistic configuration effectively addresses the challenges of ionic conductivity, interfacial stability, and dendrite suppression, offering a scalable electrolyte design strategy for next-generation solid-state lithium metal batteries.

### 2.5. Stability of Batteries

**Figure 7:**
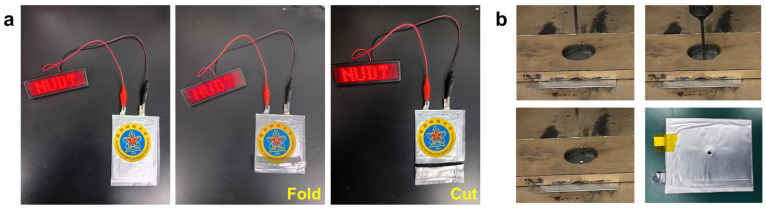
(**a**) Discharge behavior of the CGE pouch cell under normal, folded, and cut conditions. (**b**) Nail penetration test of the composite solid-state pouch cell.

To evaluate the practical reliability of the CGEs under mechanical stress, flexible pouch-type full cells were assembled using NCM811 cathodes and lithium metal anodes. The fully charged pouch cell powered an LED matrix and maintained constant brightness throughout repeated folding and partial cutting tests (Figure 7a). This operational stability indicates that the CGE membrane possesses sufficient mechanical flexibility to maintain ionic pathway continuity while preventing internal short circuits under deformation. These findings are consistent with the mechanical properties of the CGE, which exhibits a tensile strength of 1.78 MPa and an elongation at break exceeding 200 percent.

Safety characteristics were further examined via a nail penetration test conducted at a constant rate of 0.1 mm/s using a 3 mm diameter steel nail (Figure 7b). The battery maintained structural integrity without electrolyte leakage, thermal runaway, or combustion throughout the penetration process. The observed safety response is attributed to the interaction between the LLZTO fillers and the PVDF-HFP network, which forms a physical barrier against thermal and mechanical failure. Specifically, the Lewis acidic sites on the LLZTO surface contribute to the reinforcement of the polymer matrix and help mitigate localized stress during penetration. These results demonstrate the mechanical durability and fire safety of the solid-state battery system for applications in flexible electronics and energy storage.

## 3. Conclusions

In summary, this study demonstrates the synthesis of a multiscale-coupling CGE by incorporating an optimized concentration of LLZTO within a PVDF-HFP matrix to address thermal–electrochemical degradation in solid-state lithium metal batteries. LLZTO reduces the crystallinity of the polymer, while the Lewis acidic sites on its particle surface effectively anchor TFSI− anions, promoting the decoupling of Li^+^ from its primary solvation sheath. This interaction reduces the Li^+^ migration activation energy to 0.248 eV and enhances the mechanical strength of the electrolyte to 1.78 MPa. Consequently, the regulated ion flux facilitates the formation of a bimodal interphase chemistry consisting of a dense, inorganic-rich (LiF/Li_3_N/Li_2_S) SEI that suppresses lithium dendrite growth and a conformal CEI that extends the anodic stability window to 4.3 V. Enabled by this synergistic regulation, symmetric cells demonstrate stable, polarization-free operation for over 450 h at 80 °C. This work establishes that utilizing Lewis acid–base interactions at critical filler concentrations is an effective strategy to integrate efficient ion transport, mechanical robustness, and targeted interfacial passivation for advanced energy storage under extreme conditions.

## 4. Materials and Methods

### 4.1. Preparation of Gel Polymer Electrolytes

PVDF-HFP (Kynar Flex 2801, Arkema, Colombes, France) was dissolved in methyl ethyl ketone (Sinopharm Chemical Reagent Co., Ltd., Shanghai, China) and stirred at 60 °C for 1 h to form a clear polymer solution with a concentration of 6.7 wt%. Predetermined amounts of LLZTO (Shenzhen Kejing Star Technology Co., Ltd., Shenzhen, China), LiTFSI (Sigma-Aldrich Corporation, St. Louis, MO, USA), and EMITFSI (Lanzhou Institute of Chemical Physics, Chinese Academy of Sciences, Lanzhou, China) were then introduced into the solution under an inert atmosphere, while the mass ratio of PVDF-HFP:LiTFSI:EMITFSI was maintained at 5:5:7. The mixture was further stirred for 3 h until a homogeneous casting solution was obtained, followed by casting into a stainless-steel mold. After drying in a vacuum oven at 100 °C for 24 h to remove the residual solvent, the resulting membrane was obtained and denoted as GE. Before use, the membrane was punched into circular disks with a diameter of 16 mm. CGEs were prepared by following the same procedure, except that different amounts of LLZTO were incorporated, while all other preparation parameters were kept unchanged.

### 4.2. Preparation of Composite Cathode

PVDF-HFP was dissolved in NMP (Sinopharm Chemical Reagent Co., Ltd.) at 60 °C under magnetic stirring for 30 min to form a clear polymer solution with a concentration of 6.7 wt%. LiTFSI and EMITFSI were subsequently added while maintaining a mass ratio of 5:5:7, after which the resulting gel was mixed with NCM811 (Shenzhen Kejing Star Technology Co., Ltd.), conductive carbon KS-6, superconductive carbon SP, and additional NMP using a planetary ball mill for 20 min. The obtained slurry was then dried under vacuum at 110 °C for 24 h to produce the composite cathode. The final cathode contained 77 wt% active material, with an areal loading of approximately 2~3 mg cm^−2^.

### 4.3. Material Characterization

SEM (HITACHI S-4800, Tokyo, Japan) was employed to examine the morphology of the samples, while XPS (Thermo Scientific K-alpha, Thermo Fisher Scientific, Waltham, MA, USA) was conducted to probe the surface chemical-state evolution of the cathode and lithium anode. The mechanical performance of the electrolytes was evaluated using a universal testing machine (CMT6103, MTS Industrial Systems (China) Co., Ltd., Shenzhen, China) at a tensile rate of 10 mm min^−1^ with dumbbell-shaped specimens.

### 4.4. Electrochemical Measurements

Solid-state cells were assembled using the composite cathode, the lithium metal anode, and the prepared GEs or CGEs in a CR2016 coin-cell configuration. Galvanostatic charge/discharge tests were carried out on a LAND CT2001A battery test system to evaluate the cell performance. All cells were initially activated at 0.2 C for five cycles, after which they were cycled at the designated current rates within a voltage window of 2.8~4.3 V. Electrochemical measurements, including EIS, LSV, and CA, were conducted on a Metrohm Autolab PGSTAT302N electrochemical workstation at 80 °C. For EIS, the applied AC amplitude was 10 mV over a frequency range from 10^6^ to 0.1 Hz. LSV was performed at a scan rate of 0.1 mV s^−1^ over the voltage range of 0~6 V, while CA was conducted with a polarization voltage of 20 mV.

### 4.5. Computational Methods

DFT calculations were performed using the DMol3 module in Materials Studio 2023. The GGA-PBE functional, together with Grimme DFT-D dispersion correction and the DNP 4.4 basis set, was employed in all calculations. Geometry optimization for all structures was carried out at a fine quality level. The Brillouin zone was sampled at the Gamma point (1 × 1 × 1). The convergence criteria were set to 1.0 × 10^−5^ Ha for energy, 2.0 × 10^−3^ Ha Å^−1^ for maximum force, and 5.0 × 10^−3^ Å for maximum displacement.

The adsorption energy was calculated using the following formula:

*E_ads_* = *E_total_*_−_
*E_slab_*_−_
*E_molecule_* (1)

To probe the interfacial ion-transport dynamics, amorphous cell models were constructed for MD simulations. Two electrolyte systems were built for comparison: a composite electrolyte model (CGEs) containing 25 PVDF-HFP chains, 125 EMITFSI molecules, 120 LiTFSI molecules, and one LLZTO nanoparticle, and a control electrolyte model (GEs) containing 25 PVDF-HFP chains, 125 EMITFSI molecules, and 120 LiTFSI molecules. Both models were initialized with a density of 1.260 g/cm^3^.

All MD simulations were carried out using the Forcite module. The Universal force field was adopted to describe the intra- and intermolecular interactions among the polymer, ionic liquid, and lithium salt species. The initial structures were first subjected to geometry optimization to remove unfavorable steric contacts. The optimized models were then equilibrated in the NVT ensemble at 298 K for 200 ps using a Nosé thermostat, where the number of particles, system volume, and temperature were kept constant.

## Data Availability

The original contributions presented in the study are included in the article. Further inquiries can be directed to the corresponding author.

## Supplementary Materials

The following supporting information can be downloaded at: https://www.mdpi.com/article/10.3390/gels12040283/s1, Figure S1: Optical photograph of the GE film.; Figure S2: Identified elemental species in GEs and their atomic ratios (At%); Figure S3: Identified elemental species in CGEs and their atomic ratios (At%); Figure S4: Identified elemental species in cross-sectional CGEs and their atomic ratios (At%); Figure S5: SEM image and corresponding elemental mapping images of CGEs with 0.05 g LLZTO loading; Figure S6: SEM image and corresponding elemental mapping images of CGEs with 0.08 g LLZTO loading; Figure S7: SEM image and corresponding elemental mapping images of CGEs with 0.2 g LLZTO loading; Figure S8: SEM image and corresponding elemental mapping images of CGEs with 0.3 g LLZTO loading; Figure S9: SEM image and corresponding elemental mapping images of CGEs with 0.4 g LLZTO loading; Figure S10: SEM image and corresponding elemental mapping images of CGEs with 0.5 g LLZTO loading; Figure S11: Nyquist plots of the SGEs with different LLZTO contents; Figure S12: Kinetic model of GEs; Figure S13: C 1s, S 2p and N 1s XPS spectra at various sputtering depths on the Li metal surface from the long-term-cycled Li/CGE/Li symmetric cell; Figure S14: C 1s, S 2p and N 1s XPS spectra at various sputtering depths on the Li metal surface from the long-term-cycled Li/GE/Li symmetric cell; Figure S15: Galvanostatic charge–discharge (GCD) curves of the NMC811/CGE/Li cell during cycling performance tests; Figure S16: Galvanostatic charge–discharge curves of the LFP/CGE/Li cell during cycling performance tests; Figure S17: O 1s, S 2p and N 1s XPS spectra at various sputtering depths on the cycled NCM811 cathode.
