# Supplementary material for: Synthesis of Li6.4La3Zr1.4Ta0.6O12-Incorporated Composite Gel Electrolytes via Competitive Anion Anchoring for Dual-Interface Stabilization in Lithium Metal Batteries
Source: Gels. 2026 Mar 28;12(4):283. doi: 10.3390/gels12040283 (PMC13115217; doi:10.3390/gels12040283)
Supplement: Supplementary file 1 [file gels-12-00283-s001.zip › gels-4213533-supplementary.pdf]

$Li_{6.4}La_3Zr_{1.4}Ta_{0.6}O_{12}$-Incorporated Composite Gel Electrolytes via Competitive Anion Anchoring for Dual-Interface Stabilization in Lithium Metal Batteries

Jie Zhao [1], Maoyi Yi [1], Chunman Zheng [1,]* and Qingpeng Guo [1,]*

[1] College of Aerospace Science and Engineering, National University of Defense Technology, Changsha, Hunan 410073, China; zhaojie15@nudt.edu.cn; yimaoyinudt@nudt.edu.cn

* Correspondence: zhengchunman@nudt.edu.cn; qingpeng.guo@nudt.edu.cn

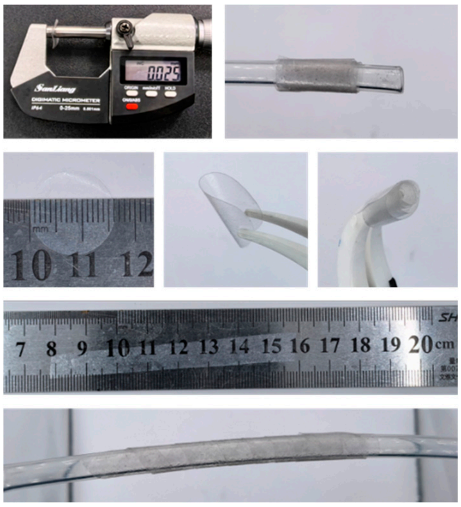

Figure S1. Optical photograph of the GEs film.

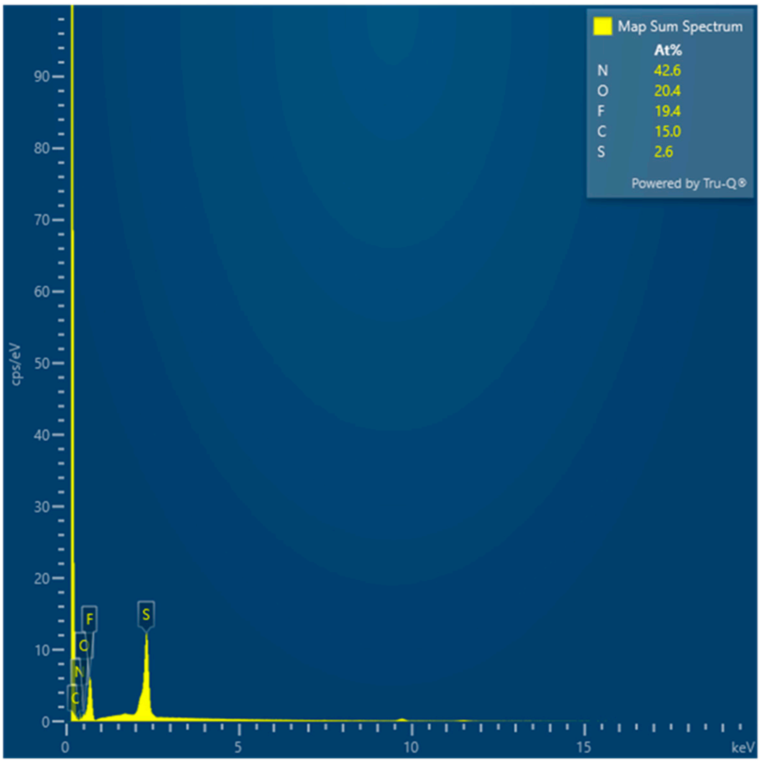

Figure S2. Identified elemental species and their atomic ratios (At%) of GEs.

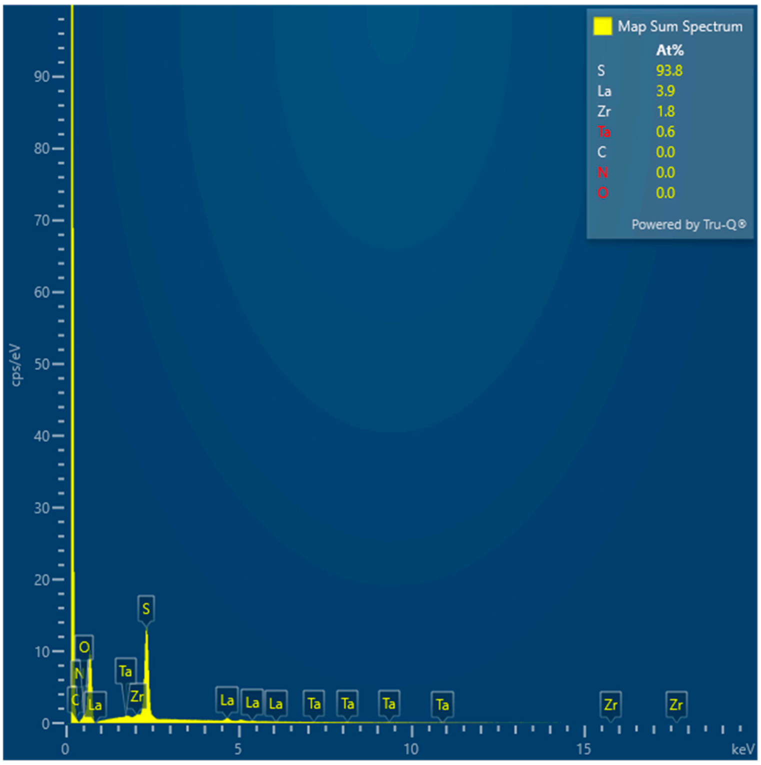

Figure S3. Identified elemental species and their atomic ratios (At%) of CGEs.

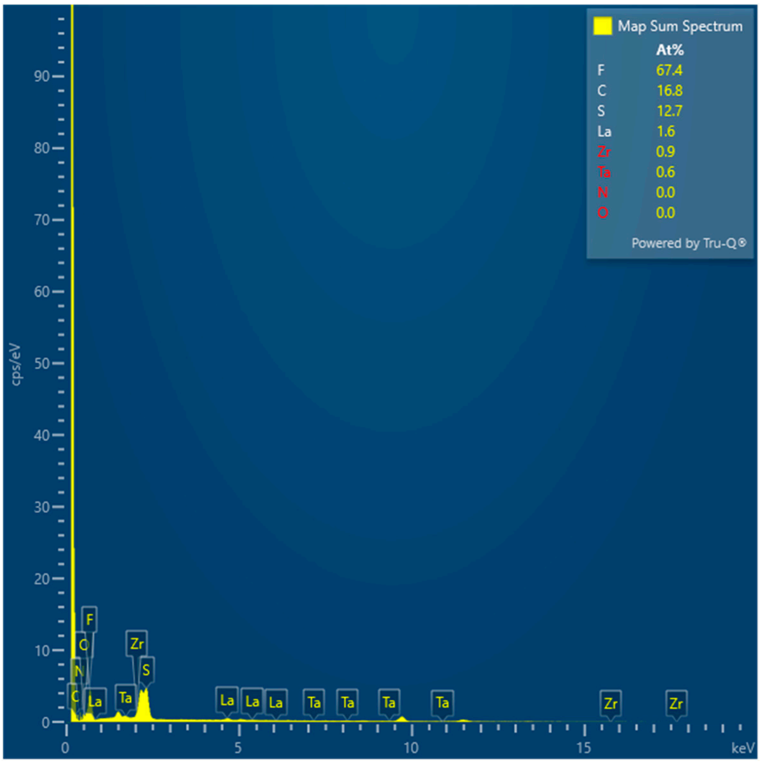

Figure S4. Identified elemental species and their atomic ratios (At%) of cross-sectional CGEs.

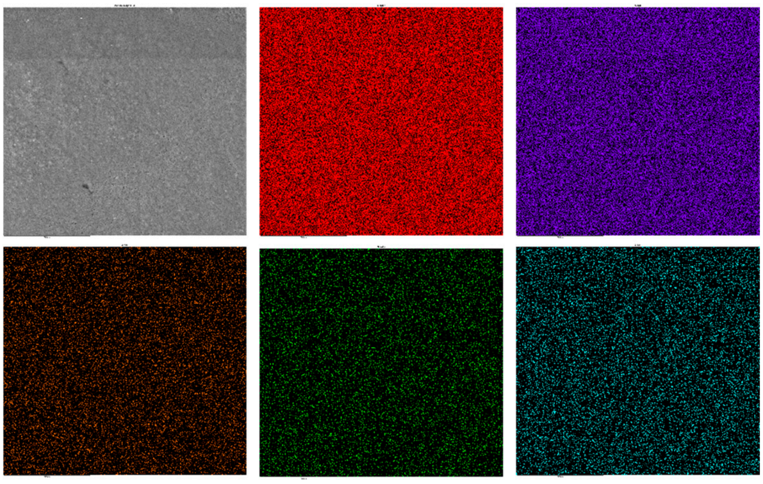

Figure S5. SEM image and corresponding elemental mapping images of CGEs with 0.05 g LLZTO loading.

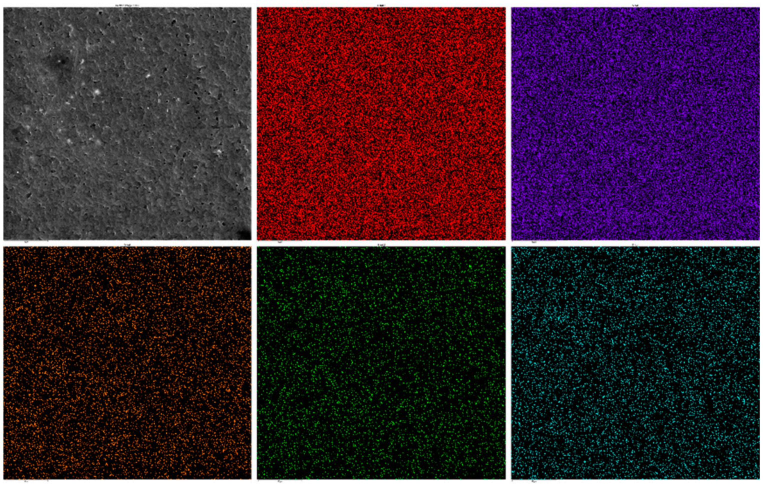

Figure S6. SEM image and corresponding elemental mapping images of CGEs with 0.08 g LLZTO loading.

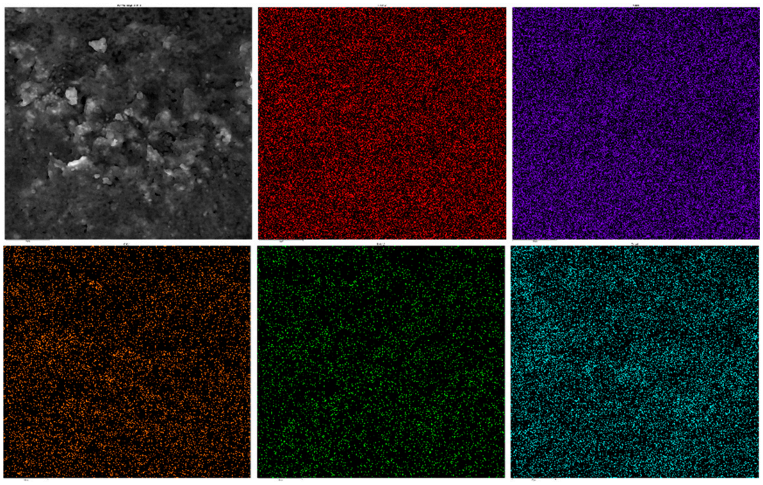

Figure S7. SEM image and corresponding elemental mapping images of CGEs with 0.2 g LLZTO loading.

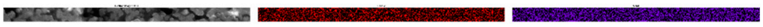

Figure S8. SEM image and corresponding elemental mapping images of CGEs with 0.3 g LLZTO loading.

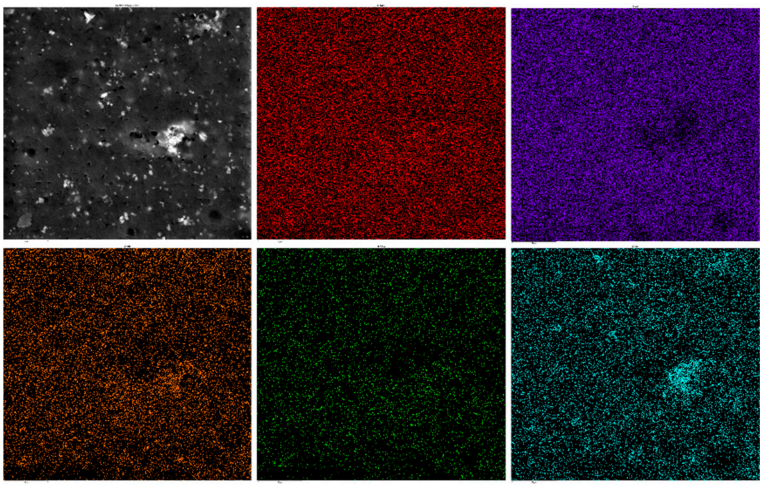

Figure S9. SEM image and corresponding elemental mapping images of CGEs with 0.4 g LLZTO loading.

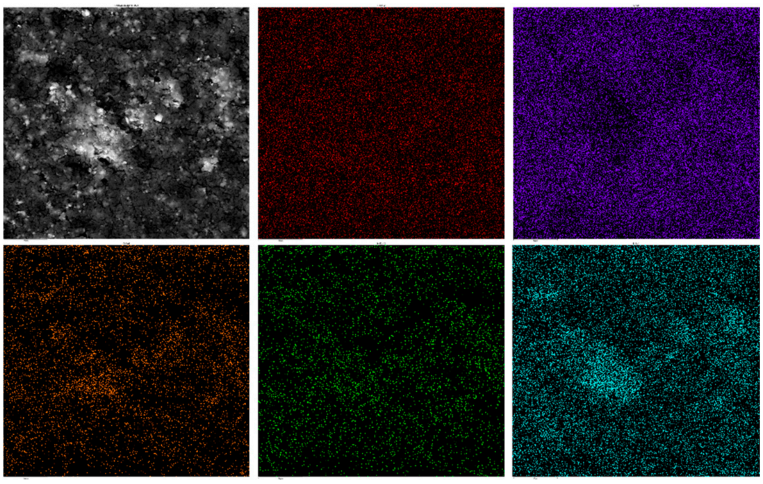

Figure S10. SEM image and corresponding elemental mapping images of CGEs with 0.5 g LLZTO loading.

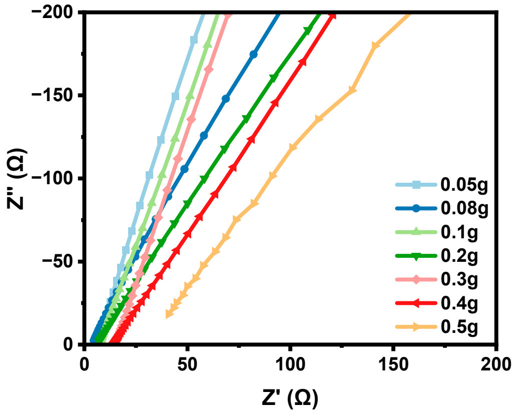

Figure S11. Nyquist plots of the SGEs with different LLZTO contents.

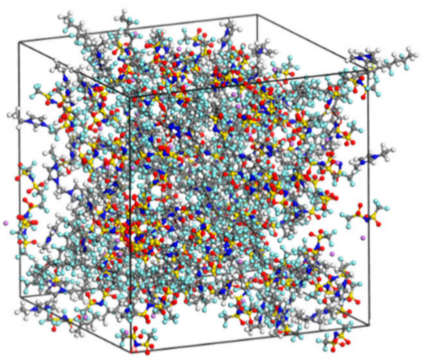

Figure S12. Kinetic model of GEs.

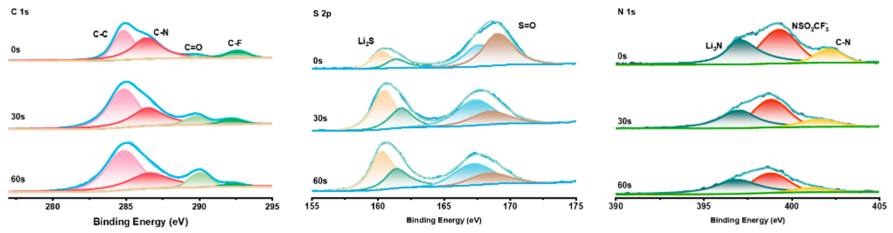

Figure S13. C 1s, S 2p and N 1s XPS spectra at various sputtering depths of the Li metal surface from the long-term cycled Li/CGEs/Li symmetric cell.

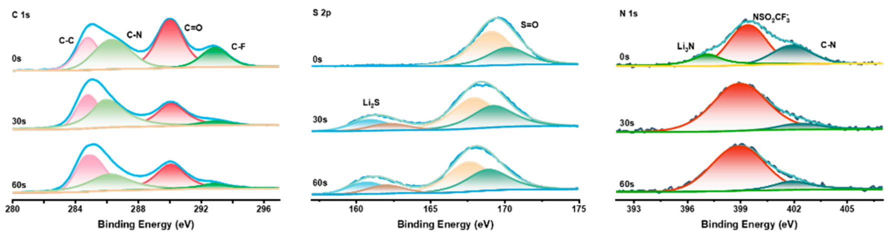

Figure S14. C 1s, S 2p and N 1s XPS spectra at various sputtering depths of the Li metal surface from the long-term cycled Li/GEs/Li symmetric cell.

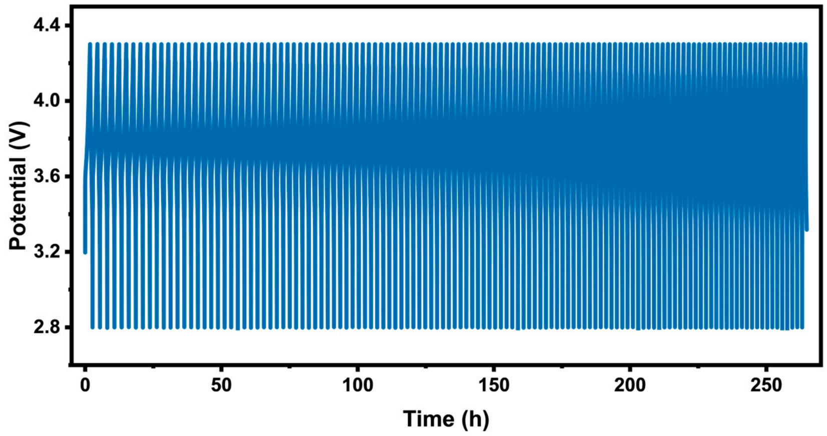

Figure S15. Galvanostatic charge-discharge (GCD) curves of the NMC811/CGEs/Li cell during cycling performance tests.

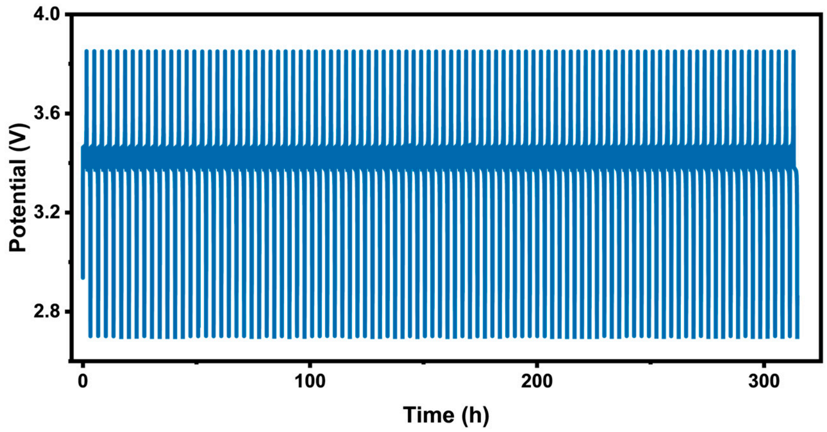

Figure S16. Galvanostatic charge-discharge curves of the LFP/CGEs/Li cell during cycling performance tests.

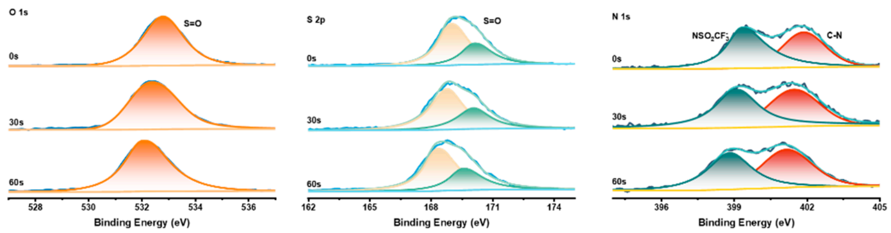

Figure S17. O 1s, S 2p and N 1s XPS spectra at various sputtering depths of the cycled NCM811 cathode.
